# Evaluation of osteomeatal complex by cone-beam computed tomography in patients with maxillary sinus pathology and nasal septum deviation

**DOI:** 10.1186/s12903-024-04272-z

**Published:** 2024-05-10

**Authors:** Burak İncebeyaz, Bengi Öztaş

**Affiliations:** Department of Dentomaxillofacial Radiology, Faculty of Dentistry, Ankara University, Ankara, Besevler 06500 Turkey

**Keywords:** Osteomeatal complex, Maxillary sinus, Nasal septum, Maxillary sinus ostium, Infundibulum

## Abstract

**Background:**

This study aimed to determine if there is a relationship between the presence of maxillary sinus pathology, nasal septum deviation and various lengths of the osteomeatal complex.

**Methods:**

A total of 223 CBCT images were included in the study. The lengths of the osteomeatal complex (maxillary sinus ostium width, infundibulum length, maxillary sinus ostium height) were analyzed. The presence of maxillary sinus pathology, nasal septum deviation, age, sex, right-left, septum deviation level, and the relationship between pathology level and all variables were evaluated.

**Results:**

The average maxillary sinus ostium width, ostium height and infundibulum length were 3.06 ± 0.70 mm, 30.10 ± 5.43 mm and 8.82 ± 1.86 mm, respectively. Ostium width was significantly higher in the healthy group than in the groups evaluated in the presence of deviation and pathology. A significant difference was found in infundibulum length only between the healthy condition and the condition evaluated in the presence of deviation. No significant difference was observed between the groups in terms of ostium height. In all groups, ostium height and infundibulum length were significantly higher in men than in women. The age group with the highest average ostium height was found in the 35–44 age group (*p* < 0.001).

**Conclusion:**

Identifying normal and abnormal conditions in the osteomeatal complex area is important for diagnosing the cause of a patient's complaint, guiding the surgical procedures to be performed, and preventing possible complications that may arise during surgical procedures.

## Introductıon

The paranasal sinuses consist of air spaces of various shapes and sizes within the surrounding bones that connect to the structure of the nasal cavity[1]. These spaces are closely related to the dentomaxillofacial region[2, 3]. The lower face, including the maxilla, is altered by the development of tooth germs and the longitudinal growth of the maxillary sinus[4].

The natural ostium of the maxillary sinus is located in the upper part of its medial wall, typically behind the midpoint of the bulla ethmoidalis. The posterior extension of the uncinate process indicates the location of the ostium[5].

As the paranasal sinuses continue to develop during childhood, patients are prone to significant anatomical variations and congenital malformations, as well as acquired infectious, inflammatory, and neoplastic diseases[6]. Understanding the normal developmental pattern and pneumatization of the paranasal sinuses as well as anatomical variations is important to evaluate sinus diseases and recommend appropriate treatment and surgical guidance[7]. Anatomic variations in the paranasal sinuses and surrounding structures are common and can cause impaired sinus drainage,leading to issues with sinus aeration and mucociliary activity, which can predispose patients to infections[8].

The osteomeatal complex (OMC) is a structure formed by the maxillary sinus ostium, infundibulum, middle meatus, ethmoid bulla and uncinate process. It serves as the final common route for drainage and ventilation of the frontal, maxillary and anterior ethmoid air cells[9]. The OMC has a narrow anatomical structure and is susceptible to narrowing in various pathological conditions[10–12].

Anatomical variations such as hyperplastic uncinate process, concha bullosa, maxillary ostium stenosis, septal deviations or nasal polyposis can lead to impaired drainage of the maxillary sinus and reduced ciliary activity. This can result in lower oxygen levels and higher carbon dioxide concentrations. Subsequently impaired ventilation, drainage, and epithelial dysfunction in the sinuses can make patients more susceptible to infections, leading to edema and mucosal hypertrophy in the OMC[13].

Cone-beam computed tomography (CBCT) is superior to traditional computed tomography in terms of its compact design, fast imaging time, low cost and low radiation dose. With these advantages, CBCT has become routinely used for imaging anatomical structures and variations in the paranasal sinuses[14].

Previous studies have assessed the impact of different anatomical conditions on the osteomeatal complex and the significance of anatomical variations in the development of rhinosinusitis[15–17]. Additionally only two reports have been made on inflammatory sinus pathologies affecting the ostium height or infundibulum lenght of the maxillary sinus[18, 19]. While there have been previous studies examining the relationship of the parameters in our study with individual maxillary sinus pathologies or anatomical variations, no studies have evaluated all three parameters together. Infundibulum length, ostium height and ostium width are crucial in providing information on both the volume of the maxillary sinus and its drainage. This study, aimed to evaluate CBCT images to determine if there was any correlation between the presence of maxillary sinus pathology or nasal septum deviation and various measurements of the OMC, such as infundibulum length, ostium height and ostium width.

## Materials and Methods

The study was carried out in accordance with the principles of the Declaration of Helsinki to include all regulations and revisions.

In this study, 292 maxillary sinus CBCT images of 223 patients were evaluated among 1651 CBCT images obtained for various reasons from patients treated at the Department of Oral, Dental and Maxillofacial Radiology of the Faculty of Dentistry between November 2016 and December 2017.

In the present study, images of male and female patients aged 15–85 years with no artifacts with septum deviation and/or maxillary sinus pathology where the maxillary sinus ostium and infundibulum completely entered the imaging field were included. Images of patients in which the ostium of the maxillary sinus did not enter the imaging area and motion or metal artifacts were not included in the study.

A total of 223 CBCT images suitable for the study criteria were used; 113 males and 110 females aged between 15 and 85 years and who met these criteria were included in the study.

### Evaluation of images

All of the CBCT images used in the study were taken with the Planmeca ProMax 3D Max (Planmeca, Promax, Finland) device. The images were obtained with 130 × 55, 130 × 90, 230 × 160, and 230 × 270 mm FOVs; 96 kVp; 5.6 and 7.8 mA; 9–12 s; 0.2 × 0.2x0.2 mm; and 0.4 × 0.4x0.4 mm voxel size. A signed consent form is obtained from each patient who will undergo radiological examination in Ankara University Faculty of Dentistry Radiology Clinic. The original software of the device, Planmeca Romexis (3.7; Planmeca, Helsinki, Finland), was used for radiographic evaluations. All the images are displayed on a 21.3-inch flat panel with a color-active matrix and thin-film transistor (TFT) medical monitor (NEC MultiSync MD215MG, München) with a resolution of 2048 × 2560 at 75 Hz and a 0.17 mm dot pitch at 11.9 bits, Germany) were viewed and evaluated. To assess intraobserver agreement, all measurements were repeated approximately 1 month after the first assessment.

**Fig. 1:**
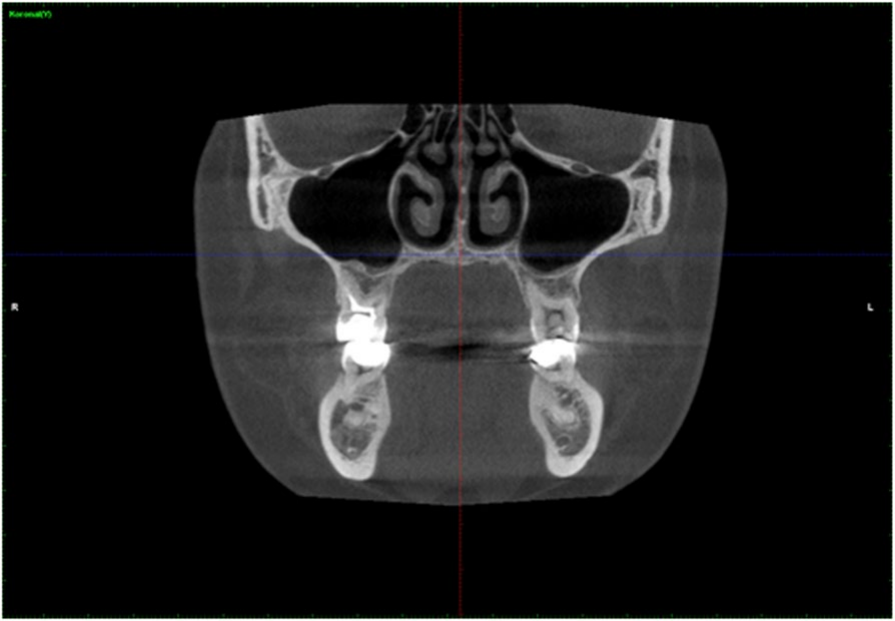
Making the nasal base parallel to the horizontal plane for standardization in CBCT coronal sections

In order to standardize the images in CBCT sections, the head position in the images was made parallel to the horizontal plane in the coronal sections of the nasal cavity floor (Fig. 1).

### Evaluation criteria used in the study

The evaluation criteria were examined under 2 main headings as qualitative and quantitative variables.

### Quantitative Variables

#### Measuring the maxillary sinus ostium width

**Fig. 2:**
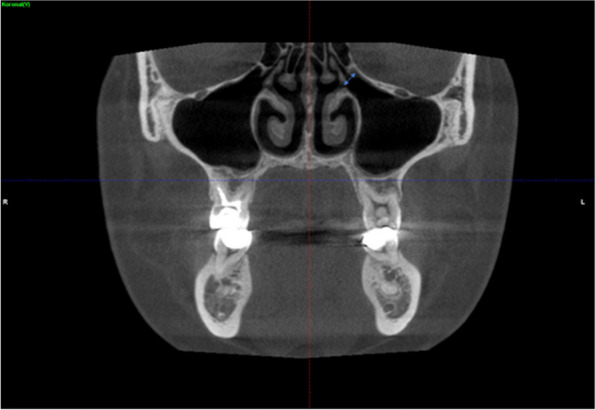
Coronal CBCT images showing the ostium width

The maxillary sinus ostium is the upper part of the medial wall of the sinus. By taking the point where the ostium starts, the widest part was determined, and the measurement was made between the bone levels from here (Fig. 2).

#### Measurement of maxillary sinus ostium height

**Fig. 3:**
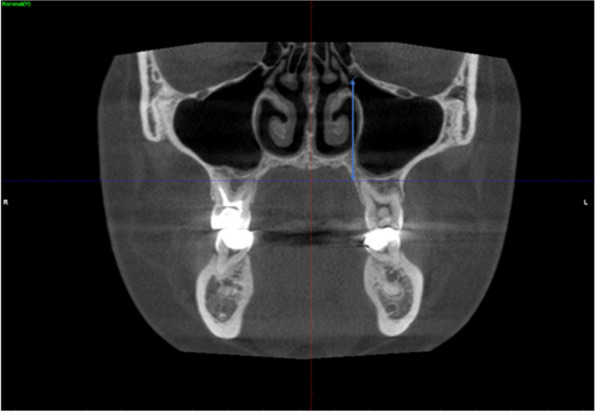
Coronal CBCT images showing the ostium height

The distance between the midpoint of the maxillary sinus ostium and the lowest bone level of the maxillary sinus was measured (Fig. 3).

#### Measurement of infundibulum length

**Fig. 4:**
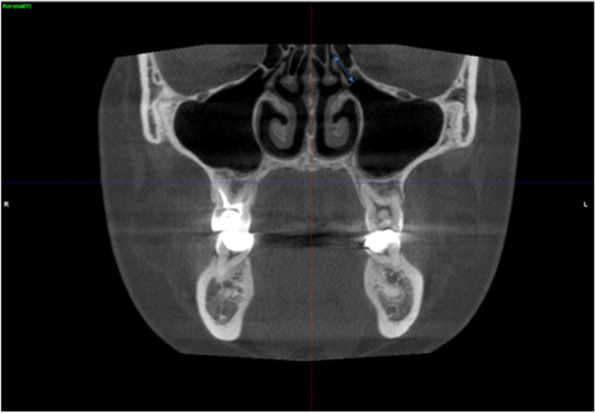
Coronal CBCT images showing the infundibulum length

The distance between the center of the ostium and the top of the uncinate process was measured (Fig. 4).

### Qualitative Variables

#### Determination of nasal septum deviation in the coronal plane

**Fig. 5:**
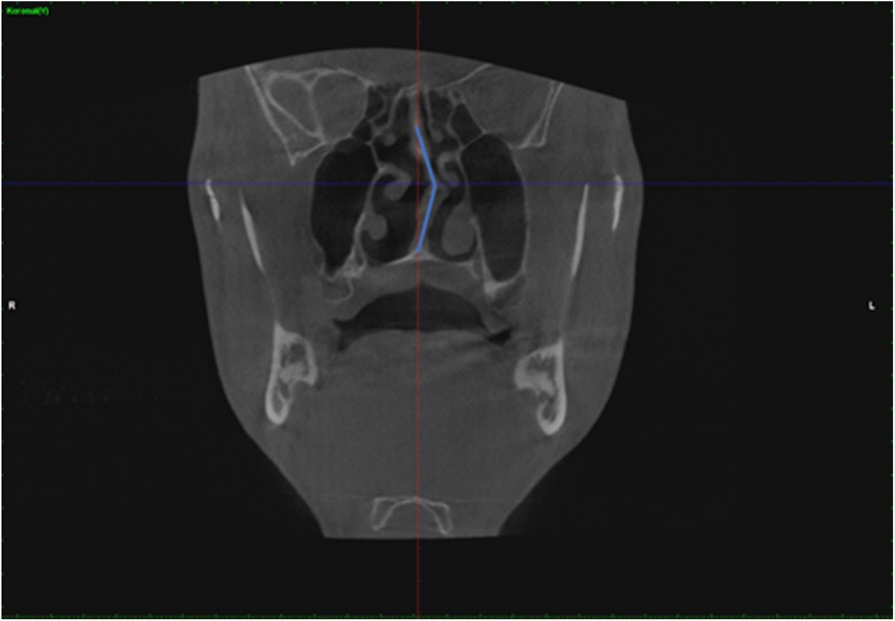
Determination of nasal septum deviation in the coronal CBCT images

The deviation angle was accepted as the angle between the linear line drawn from the maxillary spina to the crista galli and the linear line drawn from the crista galli to the most deviated part of the nasal septum. The direction of the deviation was defined by the convexity of the septal curvature (Fig. 5).

**Fig. 6:**
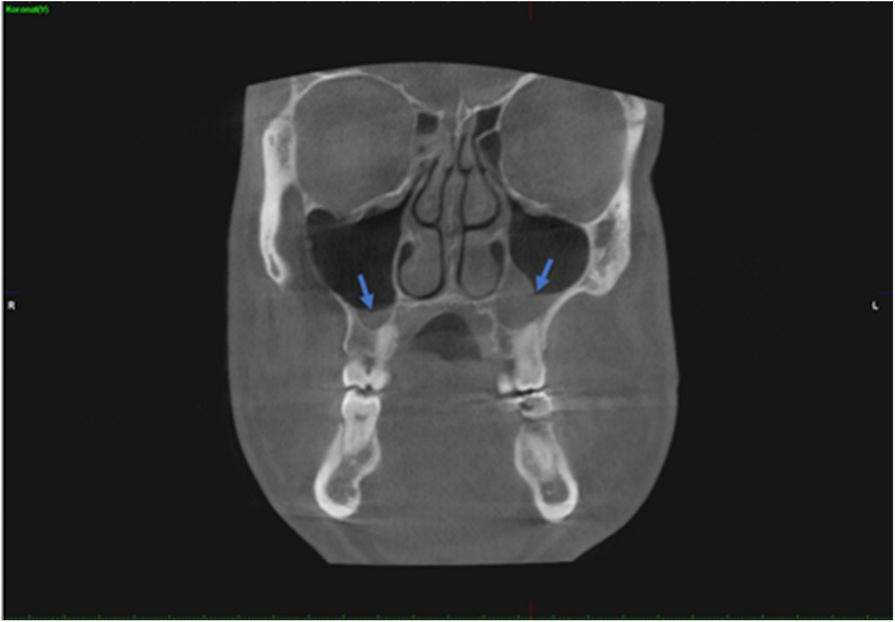
Determination of the presence of maxillary sinus pathology in the coronal CBCT images

**Fig. 7:**
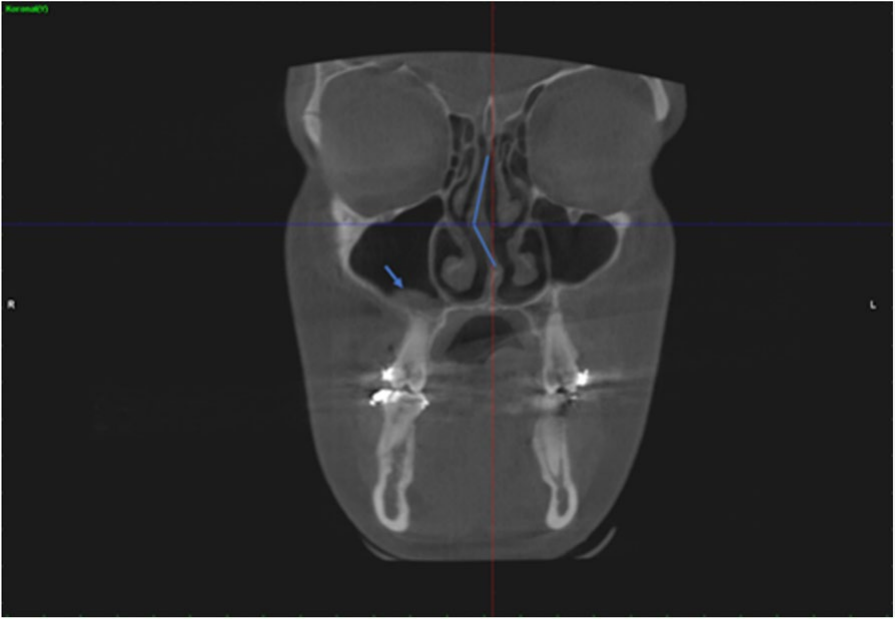
Determination of the coexistence of maxillary sinus pathology and nasal septum deviation in the coronal CBCT images

In addition, the presence of maxillary sinus pathology in the coronal plane and the coexistence of maxillary sinus pathology and nasal septum deviation were determined (Figs. 6 and 7).

### Statistical analysis

The SPSS 11.5 program was used in the analysis of the data. The mean ± standard deviation and median (minimum–maximum) were used as descriptors for quantitative variables, and the number of patients (percentage) was used for qualitative variables. To determine whether there was a difference between the two categories of qualitative variables in terms of quantitative variables, if the normal distribution assumptions were met, Student’s t test was used; if not, the Mann‒Whitney U test was used. To determine whether there was a difference between the categories of qualitative variables with more than two categories in terms of quantitative variables, if the normal distribution assumptions were met, one-way ANOVA was used; otherwise, the Kruskal–Wallis H test was used. A paired t test was used when normally distributed data was available, and the Wilcoxon signed test was used when it was not. The intraclass fit test was used to check the intraobserver fit. The statistical significance level was set at 0.05.

## Results

The study included 90 maxillary sinus CBCT images (30.8%) of patients without maxillary sinus pathology and septum deviation (Control group), 57 maxillary sinus CBCT images (19.5%) of patients with nasal septum deviation, and 84 maxillary sinus CBCT images (28.8%) of patients with sinus pathology. Additionally, there were 61 maxillary sinus CBCT images (20.9%) of patients in whom pathology and deviation were observed together. In total, 292 right-left maxillary sinus CBCT images were evaluated from 223 patients. The mean width of the maxillary sinus ostium was 3.06 ± 0.70 mm, the mean height of the maxillary sinus ostium was 30.10 ± 5.43 mm, and the mean length of the infundibulum was 8.82 ± 1.86 mm. In addition, the general distribution of CBCT images according to age, gender, septal deviation angles and pathology levels is shown in Table 1.

**Table 1:** General identifiers

Variables		
**Evaluated groups, n (%)**	Control	90 (30,8)
	Deviation ( +)	57 (19,5)
	Pathology ( +)	84 (28,8)
	Pathology and Deviation ( +)	61 (20,9)
**Ostium width (mm)**	**Mean ± SD**	3,06 ± 0,70
	**Median (Min–Max)**	3,05 (1,27–5,73)
**Ostium height (mm)**	**Mean ± SD**	30,10 ± 5,43
	**Median (Min–Max)**	30,40 (15,00–42,40)
**Infundibulum length (mm)**	**Mean ± SD**	8,82 ± 1,86
	**Median (Min–Max)**	8,60 (4,70–13,90)
**Gender, n (%)**	Male	144 (49,3)
	Female	148 (50,7)
**Age, n (%)**	< 18	11 (3,8)
	18–24	47 (16,1)
	25–34	91 (31,1)
	35–44	44 (15,1)
	45–54	32 (11,0)
	55–64	39 (13,3)
	> 65	28 (9,6)
**Septal deviation angle, n (%)**	0–5	10 (17,5)
	5–10	20 (35,1)
	10–15	16 (28,1)
	> 15	11 (19,3)
**Pathology level, n (%)**	Mucosal Thickening	41 (48,8)
	Antral Pseudocyst	34 (40,5)
	Partial Opacification	9 (10,7)

*SD* Standard deviation, *Min* Minimum, *Max* Maximum

Among the groups evaluated in Table 2, differences in ostium width, height and infundibulum length between the control group and the other groups were examined. A significant difference was found between the control group and the groups evaluated in terms of deviation, pathology, pathology and deviation in terms of ostium width (*p* < 0.001, *p* = 0.006 and *p* < 0.001, respectively). Regarding ostium height, no significant difference was found between the control group and the other groups in terms of deviation, pathology, pathology or deviation (*p* = 1,000, *p* = 0.606 and *p* = 0.158, respectively). Only a significant difference in infundibulum length was found between the control group and the group evaluated in the presence of deviation (*p* = 0.036) (Table 2).

**Table 2:** Comparison of structural differences between the bone levels for ostium width, ostium height, and ınfundibulum length of the control group and other groups between the evaluated groups

Variables		Control	Deviation	Pathology	Pathology and deviation	*p*
**Ostium width (mm)**	**Mean ± SD** **(mm)**	3,39 ± 0,67	2,91 ± 0,67	2,99 ± 0,66	2,79 ± 0,67	< 0,001^bx^ 0,006^by^ < 0,001^bz^
	**Median (Min–Max)(mm)**	3,40 (2,00–5,73)	2,88 (1,70–5,09)	3,06 (1,27–5,01)	2,83 (1,56–4,82)	
**Ostium height (mm)**	**Mean ± SD** **(mm)**	29,41 ± 5,52	29,42 ± 5,30	30,43 ± 5,78	31,29 ± 4,75	1,000^ax^ 0,606^ay^ 0,158^az^
	**Median (Min–Max)(mm)**	29,20 (17,00–42,40)	28,80 (17,60–40,80)	31,10 (15,00–41,20)	31,60 (20,00–42,00)	
**Infundibulum length (mm)**	**Mean ± SD** **(mm)**	9,20 ± 1,97	8,19 ± 1,70	9,13 ± 1,90	8,40 ± 1,59	0,036^bx^ 1,000^by^ 0,100^bz^
	**Median (Min–Max)(mm)**	8,80 (6,00–13,90)	8,20 (4,70–13,80)	8,80 (6,00–13,60)	8,10 (5,10–13,00)	

*SD* Standard deviation, *Min* Minimum, *Max* Maximum

^a^One Way ANOVA test

^b^Kruskal Wallis H test

^x^Normal-deviation

^y^Normal-pathology

^z^Normal-pathology and deviation comparisons

Table 3 shows the differences in ostium width, ostium height and infundibulum length between the general group and other groups based on sex. Overall, among all groups, males had significantly higher ostium height (*p* < 0.001) and infundibulum length (*p* = 0.034) compared to females.

**Table 3:** Comparison of structural differences between bone levels for ostium width, ostium height, and ınfundibulum length by gender, both overall and within groups

**Variables**	**Male**		**Female**		
	**Mean ± SD**	**Median** **(min–max)**	**Mean ± SD**	**Median** **(min–max)**	***p***
**General**					
** Ostium width (mm)**	3,13 ± 0,72	3,05 (1,70–5,73)	2,98 ± 0,68	3,03 (1,27–4,94)	0,090^b^
** Ostium height (mm)**	31,84 ± 5,56	32,00 (17,60–42,40)	28,41 ± 4,74	28,00 (15,00–38,80)	< 0,001^a^
** Infundibulum** **length (mm)**	9,08 ± 1,89	8,80 (4,70–13,90)	8,56 ± 1,81	8,35 (4,80–13,60)	0,034^b^
**Control group (no pathology – no devıatıon)**					
** Ostium width (mm)**	3,63 ± 0,71	3,54 (2,56–5,73)	3,28 ± 0,62	3,28 (2,00–4,94)	0,017^a^
** Ostium height (mm)**	32,92 ± 5,93	33,10 (20,50–42,40)	27,83 ± 4,55	28,00 (17,00–36,00)	< 0,001^a^
** Infundibulum** **length (mm)**	9,41 ± 2,22	8,75 (6,80–13,90)	9,11 ± 1,86	8,80 (6,00–12,40)	0,501^a^
**Devıatıon**					
** Ostium width (mm)**	3,17 ± 0,62	3,05 (2,00–5,09)	2,66 ± 0,63	2,56 (1,70–4,18)	0,001^b^
** Ostium height (mm)**	30,53 ± 6,26	30,80 (17,60–40,80)	28,34 ± 3,99	28,00 (20,00–36,00)	0,123^a^
** Infundibulum** **length (mm)**	8,60 ± 1,63	8,95 (4,70–13,80)	7,79 ± 1,69	7,90 (4,80–11,50)	0,073^a^
**Pathology**					
** Ostium width (mm)**	3,02 ± 0,70	3,05 (1,70–5,01)	2,95 ± 0,61	3,12 (1,27–4,33)	0,657^a^
** Ostium height (mm)**	31,62 ± 5,52	32,00 (18,00–41,2)	28,58 ± 5,76	28,00 (15,00–38,80)	0,017^a^
** Infundibulum** **length (mm)**	9,48 ± 1,89	9,00 (6,00–12,90)	8,58 ± 1,80	8,40 (6,00–13,60)	0,033^a^
**Pathology and devıatıon**					
** Ostium width (mm)**	2,88 ± 0,65	2,88 (1,84–4,82)	2,66 ± 0,71	2,62 (1,56–3,96)	0,226^a^
** Ostium height (mm)**	32,30 ± 4,70	32,00 (23,20–42,00)	29,73 ± 4,49	30,40 (20,00–37,20)	0,039^a^
** Infundibulum** **length (mm)**	8,63 ± 1,71	8,20 (6,10–13,00)	8,05 ± 1,34	7,90 (5,10–11,10)	0,163^a^

*SD* Standard deviation, *Min* Minimum, *Max* Maximum

^a^Student-t test

^b^Mann Whitney U test

In Table 4, the study examined the differences between age groups in terms of ostium width, ostium height, and infundibulum length. A significant difference was only found for ostium height (*p* < 0.001). The highest mean ostium height was observed in the 35–44 age group, followed by the 25–34, 45–54, 18–24, 55–64, > 65 and < 18 age groups. When paired groups with significant differences were analyzed using the Tukey post hoc test, significant differences were found between < 18 and 25–34 (*p* = 0.003), < 18 and 35–44 (*p* < 0.001), and 35–44 and 55–64 (*p* = 0.030). Additionally, a significant difference was found between the 35–44 and > 65 age groups (*p* = 0.023).

**Table 4:** Comparison of structural differences between bone levels for ostium width, ostium height, and ınfundibulum length by age groups

Age groups		Ostium width	*p*	Ostium height	*p*	Infundibulum length	*p*
< 18	**Mean ± SD(mm)**	3,15 ± 0,58	0,225^a^	24,82 ± 3,96	< 0,001^a^	8,32 ± 1,52	0,154^b^
	**Median (Min–Max)(mm)**	3,01 (2,26–4,24)		24,80 (17,00–29,60)		8,50 (5,10–10,20)	
18–24	**Mean ± SD(mm)**	2,89 ± 0,87		29,43 ± 5,60		8,51 ± 1,76	
	**Median (Min–Max)(mm)**	2,69 (1,27–5,73)		29,40 (17,60–41,60)		8,50 (6,00–12,60)	
25–34	**Mean ± SD(mm)**	3,01 ± 0,65		31,17 ± 5,53		8,66 ± 1,78	
	**Median (Min–Max)(mm)**	3,05 (1,56–4,47)		31,60 (15,00–42,40)		8,20 (4,80–13,90)	
35–44	**Mean ± SD(mm)**	3,21 ± 0,78		32,46 ± 4,93		9,52 ± 2,07	
	**Median (Min–Max)(mm)**	3,12 (1,79–5,09)		32,25 (18,80–41,20)		9,15 (6,00–13,80)	
45–54	**Mean ± SD(mm)**	2,93 ± 0,61		29,61 ± 4,03		8,48 ± 1,54	
	**Median (Min–Max)(mm)**	2,98 (1,70–4,25)		28,80 (21,20–37,20)		8,50 (6,20–11,80)	
55–64	**Mean ± SD(mm)**	3,20 ± 0,60		28,85 ± 4,94		8,74 ± 1,84	
	**Median (Min–Max)(mm)**	3,12 (1,70–4,82)		28,00 (20,50–40,40)		8,50 (4,70–12,70)	
> 65	**Mean ± SD(mm)**	3,14 ± 0,68		28,39 ± 5,86		9,41 ± 2,20	
	**Median (Min–Max)(mm)**	3,22 (1,70–4,49)		28,30 (18,00–39,60)		8,95 (6,10–13,60)	

*SD* Standard deviation, *Min* Minimum, *Max* Maximum

^a^One Way ANOVA test

^b^Kruskal Wallis H test

Table 5 shows comparisons of the variables for the side with and without septum deviation. There was a significant difference between the two sides in terms of ostium width and infundibulum length (*p* < 0.001 and *p* = 0.001, respectively). The means for ostium width and infundibulum length on the side without septum deviation were significantly higher than those on the side with septum deviation.

**Table 5:** Comparison of ostium width, ostium height, and ınfundibulum lengths between sides with and without septum deviation of the same patient

**Variables**	**Side with septum deviation**		**Side without septum deviation**		
	**Mean ± SD**	**Median** **(min–max)**	**Mean ± SD**	**Median** **(min–max)**	***p***
**Ostium width**	2,90 ± 0,67	2,88 (1,70–5,09)	3,22 ± 0,81	3,22 (0,00–5,38)	** < 0,001**^**b**^
**Ostium height**	29,42 ± 5,30	28,80 (17,60–40,80)	29,66 ± 5,40	29,21 (16,00–44,80)	0,349^a^
**Infundibulum** **length**	8,19 ± 1,70	8,20 (4,70–13,80)	9,04 ± 2,01	9,30 (4,12–14,40)	**0,001**^**a**^

^a^Paired-t test

^b^Wilcoxon Sign test

In Table 6, the differences in septal deviation angles for the sinuses were evaluated in the presence of deviation in terms of ostium width, ostium height, and infundibulum length. A significant difference was only found for ostium height (*p* = 0.008). When the paired groups with significant differences were examined using the Tukey post hoc test, significant differences were found between the 0–5° and 10–15° groups (*p* = 0.029), as well as between the 5–10° and 10–15° groups (*p* = 0.015).

**Table 6:** Comparison of structural differences between bone levels for ostium width, ostium height, and ınfundibulum length according to septal deviation angle in the group evaluated in the presence of deviation

Septal deviation angle (°)		Ostium width	*p*	Ostium height	*p*	Infundibulum length	*p*
0–5°	**Mean ± SD**	2,90 ± 0,70	0,978^a^	31,36 ± 3,22	0,008^a^	8,00 ± 2,01	0,113^a^
	**Median (Min–Max)**	2,56 (2,04–4,18)		32,00 (25,00–36,00)		8,15 (4,80–11,50)	
5–10°	**Mean ± SD**	2,93 ± 0,75		30,81 ± 4,21		7,59 ± 1,58	
	**Median (Min–Max)**	2,88 (1,84–5,09)		29,60 (24,80–39,20)		7,65 (4,70–10,50)	
10–15°	**Mean ± SD**	2,94 ± 0,60		25,71 ± 5,59		8,94 ± 1,84	
	**Median (Min–Max)**	2,90 (1,70–4,31)		25,80 (17,60–40,41)		9,10 (6,10–13,80)	
> 15°	**Mean ± SD**	2,83 ± 0,67		30,51 ± 6,03		8,36 ± 0,98	
	**Median (Min–Max)**	2,91 (1,72–4,12)		30,80 (20,00–40,80)		8,20 (6,60–9,90)	

*SD* Standard deviation, *Min* Minimum, *Max* Maximum

^a^One Way ANOVA test

As shown in Table 7, the pathological status was evaluated in the group assessed by pathology, but no significant difference was found in terms of any variable.

**Table 7:** Comparison of structural differences between bone levels for ostium width, ostium height, and ınfundibulum length according to pathology levels in the group evaluated in the presence of pathology

Variables		Mucosal thickening	Antral pseudocyst	Partial opacification	*p*
**Ostium width (mm)**	**Mean ± SD**	3,17 ± 0,66	2,85 ± 0,6	2,71 ± 0,80	0,113^b^
	**Median (Min–Max)**	3,22 (1,79–5,01)	3,01 (1,27–3,82)	2,56 (1,79–3,82)	
**Ostium height (mm)**	**Mean ± SD**	30,05 ± 5,45	30,64 ± 5,81	31,36 ± 7,56	0,801^a^
	**Median (Min–Max)**	30,60 (15,00–38,80)	31,80 (18,00–41,20)	34,20 (18,00–39,60)	
**Infundibulum length (mm)**	**Mean ± SD**	9,47 ± 1,81	8,64 ± 2,00	9,41 ± 1,67	0,097^b^
	**Median (Min–Max)**	9,60 (6,20–13,60)	8,10 (6,00–12,90)	9,00 (7,20–12,30)	

*SD* Standard deviation, *Min* Minimum, *Max* Maximum

^a^One Way ANOVA test

^b^Kruskal Wallis H test

Table 8 demonstrates whether there was a disparity between the measurements of healthy sinuses on the right and left sides. No significant difference was found between the measurements on the right and left sides for any of the variables.

**Table 8:** Comparison of structural differences in bone levels for ostium width, ostium height, and ınfundibulum length between right and left measurements in healthy condition

**Variables**	**Right**		**Left**		
	**Mean ± SD**	**Median (min–max)**	**Mean ± SD**	**Median (min–max)**	***p***
**Ostium width (mm)**	3,42 ± 0,64	3,39 (2,00–4,94)	3,36 ± 0,69	3,41 (2,28–5,73)	0,504^a^
**Ostium height (mm)**	29,31 ± 5,42	28,00 (18,40–41,60)	29,52 ± 5,68	29,60 (17,00–42,40)	0,558^a^
**Infundibulum length (mm)**	9,20 ± 2,04	8,80 (6,00–13,90)	9,20 ± 1,92	8,80 (6,20–13,00)	0,987^a^

*SD* Standard deviation, *Min* Minimum, *Max* Maximum

^a^Paired-t test

The intraobserver agreement for ostium width, ostium height, and infundibulum length was checked; and shown in Table 9. The coefficients of agreement were evaluated and found to be consist for ostium width, ostium height and infundibulum length.

**Table 9:** Intra-observer concordance results for ostium width, ostium height, and ınfundibulum length

Variables	Coefficient of fit	95% Confidence interval		*p*
		**Lower limit**	**Upper limit**	
**Ostium width**	0,989	0,987	0,992	< 0,001
**Ostium height**	1,000	0,999	1,000	< 0,001
**Infundibulum length**	0,996	0,995	0,997	< 0,001

## Discussion

The drainage of the maxillary sinus occurs towards the middle meatus through the ostium and infundibulum. These structures are located above the floor of the maxillary sinus, and the contents of the maxillary antrum are gradually directed to this area by mucociliary movement against gravity[18]. Obstruction caused by inflammation and anatomical variations in this region can disrupt mucociliary activity and increase the risk of paranasal sinus infection. Therefore, our study, focused on evaluating CBCT images to determine if there is any correlation between OMC lengths (such as infundibulum length, ostium width and ostium height) and maxillary sinus pathology or nasal septum deviation.

The ethmoid infundibulum is the airway that connects the maxillary sinus ostium and middle meatus. Any changes in drainage can lead to maxillary sinusitis[19]. Akay et al.[20] evaluated the correlation between infundibulum length, ostium height, anatomical variations in the osteomeatal complex (OMC), and sinus pathology in 204 patients (408 maxillary sinuses) using CBCT images. They found no significant results for infundibulum length and ostium height in relation to sinus pathology, similar to our study. They also found no significant relationship between nasal septal deviation and infundibulum length or ostium height. In our study, we found a significant difference in infundibulum length between healthy conditions and deviation status (*p* = 0,036), although there was no significant difference in ostium height. Capelli et al.[21] also studied the impact of the distance of the ostium from the floor of the maxillary sinus and the length of the ethmoid infundibulum on initial treatmentbut did not find significant results. It is believed that a longer ethmoid infundibulum may thicken the maxillary sinus mucosa, complicating drainage as it requires drainage to travel a longer distance to the middle meatus. While ostium obstruction may be related to ethmoid infundibulum length, the infundibulum could be one of the factors contributing to obstruction, though not the sole factor.

Bayrak et al.[22] investigated maxillary sinus pathologies (MSPs) and the potential relationships of these pathologies with the dimensions of the maxillary sinus ostium, finding no significant differences. This lack of difference may be attributed to overlooking various factors that could impact ostium width, such as septum deviation. Khojastepour et al.[23] reported that the correlation between mucosal thickening of the maxillary sinus and the size of the sinus ostium was not statistically significant, unlike in our study. They suggested that maxillary sinusitis might be a primary condition rather than a mechanical obstruction of the sinus ostium. However, maxillary sinusitis could potentially initiate first or arise due to various anatomical variations and conditions. Sandhu et al.[24] focused on various anatomical variations that may affect the maxillary sinus ostium in their research. They examined whether the maxillary sinus ostium should be occluded rather than focusing solely on its size. Among the 21 patients with nasal septum deviation in their study, 76.2% had an obliterated OMC, while the remaining patients exhibited a patent OMC. Structural changes occurring on the side of the deviation could lead to ostium patency loss and subsequent OMC disease. Moreover, an increase in septal deviation might raise the incidence of sinus disease by obstructing the OMC in the direction of septal angulation. Kulekci et al.[25] examined the relationship between mucosal thickening and maxillary sinus ostium width in their study but did not yield a significant result. The authors attributed this statistically insignificant distinction to the difference in ostium size solely between the bone levels and suggested that the mucosal component should also be considered as a limitation.

Carmelli et al.[26] evaluated the relationship between maxillary sinus inferior mucosal thickening and maxillary sinus obstruction. The basic assumption of their study is that maxillary sinus ostium obstruction significantly increases the risk of developing sinusitis. Consistent with these findings, our study also found that the ostium width was narrower in patients with maxillary sinus pathology. Additionally, Kato et al.[27] showed that obstruction of the maxillary sinus ostium is responsible for most cases of maxillary sinusitis.

In their study, Alkire et al.[28] examined sinonasal anatomical variants that may predispose patients to recurrent acute rhinosinusitis. The present study found that patients with recurrent acute rhinosinusitis had significantly smaller mean infundibular widths compared to control patients. These findings suggest that anatomy may play a role in the pathogenesis of recurrent acute rhinosinusitis.

Anatomical variations in the region of the osteomeatal complex, including nasal septal deviation, the presence or absence of concha bullosa, the shape of the uncinate process, and the presence or absence of Haller cells, are known to affect sinus ventilation and are associated with the development of rhinosinusitis. Gencer et al.[29] aimed to determine the possible role of nasal septal deviation in determining maxillary sinus volume and its relationship with the development of maxillary sinusitis. In the present study, mild and moderate septal deviations had no significant effect on maxillary sinus volume or sinusitis, while severe deviations had a significant effect on these parameters. According to the study by Elahi et al.[30], patients with increased nasal septal deviation were associated with a higher incidence of OMC obstruction. The side where the deviation occurs undergoes compensatory structural changes, thus causing a loss of ostium patency and a corresponding disease in the MSO. Additionally, increased septal deviation may lead to an increase in the incidence of sinus disease by causing obstruction in the septal angulation direction of the MSO.

In many studies in the literature, the impact of sex on the osteomeatal unit has been investigated. Bayrak et al.[22] did not find a statistically significant difference in ostium width according to sex. Akay et al.[20] reported statistically significant differences in ostium height based on sex, but they found no significant difference in infundibulum length. Similar to our study, infundibulum length was higher in males compared to females, although not significantly, and ostium height was also higher in males. Carvalho et al.[18] found significantly higher ostium height in males compared to females, which aligns with our results. This is supported by the fact that males generally have a greater physical height, which is reflected in the size of their sinus structures. Our findings also revealed that lower ostium height, infundibulum length and a healthy appearance were more prevalent in females than in males, indicating that easier drainage may occur with shorter ostium height and infundibulum. Teke et al.[31] examined 127 patients’ paranasal CT images and found that the height, width and length of the maxillary sinus were significantly larger in males than in females.

The highest mean ostium height was found in the 35–44 age group, followed by the 25–34, 45–54, 18–24, 55–64, > 65 and < 18 age groups. The lower ostium height in older ages is attributed to the decrease in bone level and atrophic changes in the mucosa with age. Additionally, since the maxillary sinus is still in the developmental stage under the age of 18, the ostium height is observed to be low in that age range.

In our study, we examined differences in pathology levels in the maxillary sinuses based on ostium width, ostium height and infundibulum length. Contrary to expectations, no significant differences were found any of these variables (*p* = 0.113, *p* = 0.801 and *p* = 0.097, respectively). Limitations of the study include the exclusion of total opacification and insufficient data in the group on this subject. It is possible that the results of these studies may vary in the future with additional data.

In terms of diagnosis and treatment, radiographic examinations performed in conjunction with clinical examination of the sinonasal region are complementary to clinical findings. Direct radiographs, CT, magnetic resonance imaging (MRI), ultrasonography (USG), panoramic radiography and CBCT can all be used in diagnosis of the sinonasal region. As an alternative to computed tomography, which is considered the gold standard in evaluating the anatomy of the sinonasal region and any variations that may be present, CBCT is frequently preferred in dental practice. CBCT provides cross-sectional imaging and offers advantages such as lower radiation dose, equal image quality and fewer metal artifacts compared to traditional computed tomography. It is a very useful method in evaluating paranasal sinus anatomy and pathologies.

In conclusion, obstruction caused by inflammation and anatomical variations in the OMC, a narrow region, disrupts mucociliary activity and ventilation of the sinuses, forming the basis for paranasal sinus infection. Therefore, it is important to identify normal and abnormal conditions in this region to diagnose the cause of a patient's complaint, guide treatment plans and surgical procedures, and prevent possible complications during surgery. Variations such as Haller cell, agger nasi, septation in the maxillary sinus, and maxillary sinus accessory ostium were not included in our study. Considering that these factors could impact our measurements, evaluating them in future studies may yield more significant results. To address contradictory findings in the literature, more comprehensive studies are still needed to better understand how sinonasal anatomy and variations affect the etiology of chronic rhinosinusitis.

## Data Availability

The datasets generated and/or analyzed during the current study are not publicly available for patient confidentiality and ethics reasons but are available from the corresponding author upon reasonable request.
